# Enormous adsorption of methyl blue onto Ni_x_Mg_y_Cu_(1-x-y)_Fe_2_O_4_ magnetic nanoparticles synthesized by a rapid combustion method

**DOI:** 10.3389/fchem.2026.1912403

**Published:** 2026-07-27

**Authors:** Xiaofang Zhu, Zhenghui Ge, Zhou Wang, Xuejie Fu, Zhan-ao Wu

**Affiliations:** 1 Danyang Maternal and Child Health Hospital, Zhenjiang, China; 2 The People’s Hospital of Danyang, Affiliated Danyang Hospital of Nantong University, Zhenjiang, China; 3 College of Vanadium and Titanium, Panzhihua University, Panzhihua, China; 4 Suzhou Research Center of Medical School, Suzhou Hospital, Affiliated Hospital of Medical School, Nanjing University, Suzhou, China; 5 Zhenjiang 359 Hospital, Zhenjiang, China

**Keywords:** adsorption, methyl blue, Ni_0.1_Mg_0.8_Cu_0.1_Fe_2_O_4_ magnetic nanoparticles, rapid combustion, synthesis

## Abstract

**Introduction:**

Dye wastewater poses serious threats to human health and ecosystem stability worldwide. Therefore, developing effective treatment strategies has become increasingly important.

**Methods:**

In this work, a rapid combustion approach was employed to fabricate Ni_x_Mg_y_Cu_(1-x-y)_Fe_2_O_4_ magnetic nanoparticles (MNPs) with high structural homogeneity, and characterized by several methods such as XRD, TEM, VSM, XPS, BET, and FTIR to identify for attaining a reduced particle size, and Ni_0.1_Mg_0.8_Cu_0.1_Fe_2_O_4_ MNPs with the average particle size of 17.4 nm and the saturation magnetization of 4.73 emu/g were employed due to their enhanced magnetic properties calcined at 400 °C for 2 h with ethanol of 25 mL.

**Results:**

Under initial methyl blue (MB) concentrations ranging from 800 to 1,100 mg/L, the adsorption kinetics were well described by the pseudo-second-order model, indicating that chemisorption was the dominant rate-limiting mechanism. Among the evaluated isotherm models, the Freundlich equation exhibited the best correlation with experimental data (R^2^ = 0.9893), implying a heterogeneous surface adsorption mechanism involving multiple molecular layers. Furthermore, after five consecutive adsorption–desorption cycles, the nanoparticles retained approximately 90.7% of their initial adsorption capacity, demonstrating the robust reusability and structural stability.

**Discussion:**

Ni_0.1_Mg_0.8_Cu_0.1_Fe_2_O_4_ MNPs revealed excellent stability, enormous adsorption capacity and rate of MB, and commendable recycle, which highlighted the potential of Ni_0.1_Mg_0.8_Cu_0.1_Fe_2_O_4_ MNPs as a candidate for wastewater treatment.

## 1 Introduction

The scarcity of water resources has become a critical global issue due to the release of toxic wastewater from industrial activities (Liu et al., 2026; Mola et al., 2025; Nie et al., 2026). These discharges, containing harmful substances such as organic dyes (Asai and Tapadia, 2026; Zheng et al., 2026), degrade the quality of surface and groundwater (Halligudra et al., 2022b; Halligudra et al., 2025). The rising volume of inadequately treated wastewater has exacerbated contamination of vital water bodies. This contamination poses serious environmental and health risks, as the ingestion of contaminated water causes acute and chronic diseases in both aquatic organisms and humans (Chen et al., 2026; Liang et al., 2025). Water pollution threatens human health and ecosystem stability worldwide (Mudike et al., 2022; Paramesh et al., 2021). Industrial growth and population expansion continue to increase pollutant discharge into rivers, lakes, and oceans (Patel et al., 2026; Wang and Shafieezadeh, 2025), worsening contamination and reducing clean water availability (Ikram et al., 2025; Zhang et al., 2024). Industrial dyes and organic wastewater have harmed fragile ecosystems, exacerbating the water scarcity crisis (Chikkanayakanahalli Paramesh et al., 2021; Halligudra et al., 2026; Sriramoju et al., 2021). As a result, the development of effective strategies for mitigating water pollution and conserving water resources became an urgent global priority (Guo et al., 2025; Khan et al., 2024).

Among various chemical, physical, and biological techniques explored to combat water pollution, adsorption has been widely recognized for its operational simplicity, broad applicability, low energy consumption, and limited production of secondary pollutants (Lotfy and Basta, 2026; Song et al., 2025). Adsorption involves the binding of contaminants to the surface of adsorbent materials, thereby facilitating their removal from water (Ciğeroğlu et al., 2024; Liu Z. G. et al., 2025). The unique performance and efficiency of biosorbents and magnetic adsorbents make them viable alternatives to conventional adsorbents, particularly for water purification (Shukla et al., 2024). Among these, magnetic nanoparticles (MNPs) have garnered significant attention as highly effective adsorbents for dye removal from wastewater due to their small size (Zeng H. J. et al., 2026), high surface area, biodegradability (Nguyen et al., 2025), facile separation under an applied magnetic field (Halligudra et al., 2022a) and biocompatibility (Bulin et al., 2024). These characteristics enable MNPs to interact efficiently with pollutants, thereby providing enhanced removal capacities compared to traditional adsorbents (Devi et al., 2025).

Water pollution is a pressing global issue, and effective treatment methods are crucial. One major challenge with traditional MNPs used in water treatment is their recovery after use (Meena et al., 2025; Phumsathan et al., 2025). After adsorbing contaminants, these MNPs can be difficult to separate from the water, which may lead to potential loss of material and further contamination (Anthony et al., 2025; Wang et al., 2024). The magnetic properties of MNPs allow for their easy recovery from water via an applied magnet, facilitating rapid and efficient separation and making the process more sustainable and cost-effective (Houser et al., 2024; Liu R. M. et al., 2025).

Various methods for preparing magnetic nanomaterials have been explored, including chemical precipitation (Karkhaneh and Marandi, 2024), hydrothermal (Fagundes et al., 2025; Mohamed et al., 2024), sol-gel (Shooli et al., 2024), co-precipitation (Ibrahim et al., 2017; Rakhsha et al., 2025), solid-state reaction (Mummadi and Shaik, 2024), precipitation and hydrothermal (Mohamed and Abu-Dief, 2020; Mohamed et al., 2019), and rapid combustion (Zhao S. X. et al., 2024). In contrast, the rapid combustion method offers a more efficient, cost-effective, and simplified approach for producing high-quality MNPs with short preparation times and reliable stability. Therefore, the rapid combustion method was employed to prepare Ni_0.1_Mg_0.8_Cu_0.1_Fe_2_O_4_ MNPs. Compared with the only reported leaching–coprecipitation–calcination method for preparing Ni–Mg–Cu ferrite nanomaterials in the literature (Han et al., 2019; Han et al., 2020), the calcination time was shorted from 12 h to 2 h, the calcination temperature was decreased from 1,000 °C to 400 °C, the energy consumption was reduced, and the equipment requirements were also reduced, thereby improving the safety of the preparation process.

Therefore, in this work, Ni_0.1_Mg_0.8_Cu_0.1_Fe_2_O_4_ MNPs were synthesized using rapid combustion method and employed as adsorbents for the removal of dyes from aqueous solutions, with MB serving as the model contaminant. The adsorption behavior of MB on Ni_0.1_Mg_0.8_Cu_0.1_Fe_2_O_4_ MNPs was systematically investigated.

## 2 Experimental

### 2.1 Synthesis and characterization of Ni_x_Mg_y_Cu_(1-x-y)_Fe_2_O_4_ MNPs

Ni_x_Mg_y_Cu_(1-x-y)_Fe_2_O_4_ MNPs were synthesized using a rapid combustion technique. The elemental composition significantly affects the performance of nanomaterials (Alsaedi et al., 2026). Therefore, the contents of Ni, Mg, Cu, and Fe, together with their corresponding magnetic properties, were investigated based on previous studies (Mohamed et al., 2022). In a typical synthesis, stoichiometric quantities of analytical grades nickel nitrate (Ni(NO_3_)_2_·6H_2_O), magnesium nitrate (Mg(NO_3_)_2_·6H_2_O), and copper nitrate (Cu(NO_3_)_2_·3H_2_O) were dissolved in various volumes of anhydrous ethanol (25 mL, 35 mL, 45 mL, 55 mL, and 65 mL) according to the compositions of Ni_x_Mg_y_Cu_(1-x-y)_Fe_2_O_4_ MNPs with x = 0.1 and y ranging from 0.1 to 0.8 and without pH adjustment. The mixture was then transferred into a ceramic crucible and combusted under aerobic conditions, leading to a highly exothermic, self-maintaining reaction. After the flame extinguished, a porous gel-like intermediate product was remained within the crucible. The intermediate material was subjected to calcination for 2 h at temperatures of 400 °C, 500 °C, 600 °C, 700 °C, and 800 °C using a heating rate of 3 °C/min from room temperature, with the furnace temperature gradually raised at a heating ramp of 3 °C/min. The organic impurities were removed in this process and facilitated the crystallization of the spinel structure. After naturally cooling to room temperature, the resulting calcined material was ground into a fine powder using an agate mortar under dry conditions, yielding fine Ni_0.1_Mg_0.8_Cu_0.1_Fe_2_O_4_ MNPs in powder form.

The crystalline structure of Ni_x_Mg_y_Cu_(1-x-y)_Fe_2_O_4_ was systematically probed. Specifically, structural identification was conducted via X-ray diffraction (XRD) analysis with a Rigaku D/max 2500 PC equipped with Cu-Kα radiation and X-ray photoelectron spectroscopy (XPS). A combination of SEM and TEM was utilized to observe the material’s morphology and determine its elemental composition. The magnetic behavior of the sample was subsequently evaluated with an ADE DMS-HF-4 vibrating sample magnetometer (VSM), and the specific surface area was examined by the Brunauer-Emmett-Teller (BET) method.

### 2.2 MB removal using Ni_0.1_Mg_0.8_Cu_0.1_Fe_2_O_4_ MNPs

To investigate the adsorption kinetics, multiple tests were performed by exposing the Ni_0.1_Mg_0.8_Cu_0.1_Fe_2_O_4_ MNPs to MB solutions featuring starting concentrations that ranged between 800 mg/L and 1,100 mg/L.

The experimental procedure was as follows, 5 mg of Ni_0.1_Mg_0.8_Cu_0.1_Fe_2_O_4_ MNPs were placed into each of the 12 centrifuge tubes, followed by the addition of a designated MB solution. The adsorption contact times were set at 10, 20, 30, 40, 50, 60, 80, 100, 120, 140, 160, and 180 min. After each designated contact time, the MNPs were separated from the suspension using an external magnetic field. The concentration of MB remaining in the liquid supernatant was then measured using UV–Vis spectrophotometer. The MB concentration was quantified according to a pre-established calibration curve relating absorbance to concentration. The capacity of the MNPs for MB was calculated based on Equation 1 (Salari et al., 2025).

$$Q_{e}=\frac{\left(C_{0}-C\right)V}{M}$$ (1)

where Q_e_ is the equilibrium adsorption capacity, representing the amount of MB adsorbed per gram of Ni_0.1_Mg_0.8_Cu_0.1_Fe_2_O_4_ MNPs. The initial and equilibrium MB concentrations were represented by C_0_ and C, V indicates the volume of used MB solutions utilized, while M represented the mass of Ni_0.1_Mg_0.8_Cu_0.1_Fe_2_O_4_ MNPs.

The equilibrium adsorption experiments were conducted following a protocol similar to that used in the kinetic studies, with each suspension agitated for 24 h using MB solutions with ten different initial concentrations. Equilibrium adsorption capacity was determined through Equation 1, while the characteristics of adsorption were assessed by applying the Langmuir, Freundlich, and Temkin isotherm models.

In the investigation of pH effect on the adsorption of MB on Ni_0.1_Mg_0.8_Cu_0.1_Fe_2_O_4_ MNPs, the pH value was adjusted by adding 0.1 M hydrochloric acid or 0.1 M sodium hydroxide solution into MB solutions. The recycling performance of Ni_0.1_Mg_0.8_Cu_0.1_Fe_2_O_4_ MNPs was systematically evaluated. In each experiment, 25 mg of Ni_0.1_Mg_0.8_Cu_0.1_Fe_2_O_4_ MNPs were introduced into 30 mL of a MB solution, initially concentrated at 900 mg/L, with 3 h of exposure while stirring constantly. The percentage of MB decolorization was quantified before the adsorbent was magnetically separated for regeneration. The used MNPs were then separated, calcined at 400 °C for 2 h to restore their adsorption capacity, and subjected to subsequent adsorption cycles under identical conditions. Adsorption capacity was assessed through three parallel experiments to ensure reproducibility. The recycling performance was further assessed through determination of the MB elimination effectiveness across multiple cycles using Equation 2 (Ci et al., 2025).

$$R=\frac{C_{0}-C_{R}}{C_{0}}\times 100\%$$ (2)

where, the removal efficiency, symbolized by R, quantified the extent of MB removal, while C_0_ was the initial and C_R_ was the final concentration of MB.

### 2.3 Thermodynamic study of MB adsorption

In the thermodynamic study, 25 mg of Ni_0.1_Mg_0.8_Cu_0.1_Fe_2_O_4_ MNPs were suspended into 10 mL of a 900 mg/L MB solution inside a 50 mL centrifuge tube to investigate the adsorption behavior. Adsorption experiments took place at 10 K intervals across the 303 K–323 K temperature range using water baths oscillator to ensure homogeneous mixing. The suspensions were shaken for 3 h at each temperature. After reaching equilibrium, the mixture underwent centrifugation to isolate the MNPs from the supernatant. The remaining MB levels in the supernatant were subsequently assessed using ultraviolet-visible spectrophotometry. Thermodynamic metric were derived from Equations 3–5 (Hassan et al., 2025), allowing detailed assessment of sorption.

$$K_{d}=\frac{q_{e}}{C_{e}}$$ (3)

$$\Delta G=-2.303RT\log_{10}\left(K_{d}\right)$$ (4)

$$\ln\left(K_{d}\right)=\frac{\Delta S}{R}-\frac{\Delta H}{RT}$$ (5)

where, K_d_ (g/L) was evaluated alongside essential thermodynamic properties; ΔS (J/(mol/K)) was enthalpy change; and △H (kJ/mol) was entropy change; ΔG was the standard Gibbs free energy change (kJ·mol^−1^); R was the universal gas constant (8.3144 J·mol^−1^·K^−1^); T signified absolute temperature (K); C_e_ (mg/L) and q_e_ (mg/g) denoted the MB concentration in the solid phase and liquid phase.

## 3 Results and discussion

### 3.1 Characteristic of Ni_x_Mg_y_Cu_(1-x-y)_Fe_2_O_4_ MNPs

**FIGURE 1:**
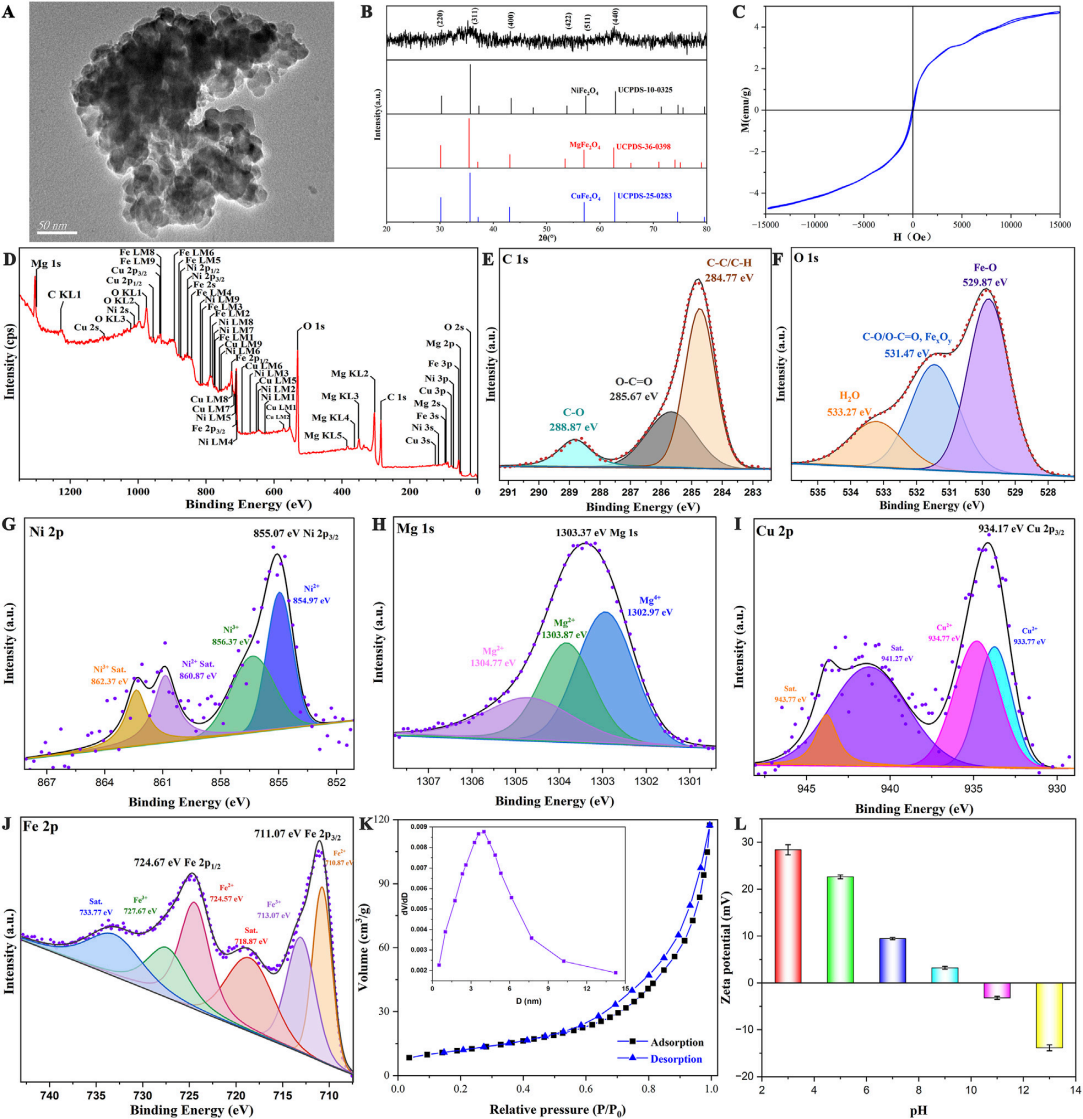
TEM micrograph **(A)**, XRD pattern **(B)**, hysteresis loops **(C)**, XPS spectra: survey scan **(D)**, C 1s **(E)**, O 1s **(F)**, Ni 2p **(G)**, Mg 1s **(H)**, Cu 2p **(I)**, Fe 2p **(J)**, nitrogen adsorption-desorption isotherms **(K)** and the pore size distribution (inset of **(K)**), and zeta potential **(L)** of Ni_0.1_Mg_0.8_Cu_0.1_Fe_2_O_4_ MNPs calcined at 400 °C for 2 h with 25 mL absolute alcohol.

The preparation of Ni_0.1_Mg_0.8_Cu_0.1_Fe_2_O_4_ MNPs was performed at 400 °C for 2 h, employing 25 mL of absolute alcohol as the solvent following by characterization using multiple techniques (shown in Figure 1). The morphology of the Ni_0.1_Mg_0.8_Cu_0.1_Fe_2_O_4_ system was investigated using TEM, as shown in Figure 1A, which showed spherical nanoparticles with an average diameter of 20.8 nm. X-ray diffraction analysis (Figure 1B) elucidated the sample’s crystal structure. Furthermore, the crystalline structure of the sample was established through the XRD pattern presented in Figure 1B, showing diffraction peaks corresponding to standard PDF cards of NiFe_2_O_4_ (JCPDS 10-0325), MgFe_2_O_4_ (JCPDS 36-0398), and CuFe_2_O_4_ (JCPDS 25-0283). Distinct diffraction peaks at 2θ values of 30.1°, 35.5°, 43.1°, 57.0°, and 62.6° were indexed to the (220), (311), (400), (511), and (440) planes, respectively, confirming the spinel crystal structure. The average crystallite size, estimated via the Scherrer equation, was found to be 17.4 nm, which was assigned to the with the TEM observation. The Ni_0.1_Mg_0.8_Cu_0.1_Fe_2_O_4_ MNPs exhibited distinctive magnetic properties compared with other compositional variants. The hysteresis loops (Figure 1C) revealed a saturation magnetization (M_s_) of 4.73 emu/g. This relatively low M_s_ value highlighted the unique magnetic behavior of Ni_0.1_Mg_0.8_Cu_0.1_Fe_2_O_4_, which was attributed to its nanostructured characteristics and crystalline structure (Zhao et al., 2025). In order to further confirm the composition of Ni_0.1_Mg_0.8_Cu_0.1_Fe_2_O_4_, XPS technique was employed to analyse their elements. The full survey scan spectrum was displayed in Figure 1D, as the photoelectron peaks of C, O, Ni, Mg, Cu, and Fe were presented, indicating the formation of Ni_0.1_Mg_0.8_Cu_0.1_Fe_2_O_4_ MNPs. The high-resolution spectra of C 1s, O 1s, Ni 2p, Mg 1s, Cu 2p, and Fe 2p were further analyzed. Figure 1E showed C 1s splitting peaks corresponded to C-C/C-H, O-C=O, and C-O at the binding energy of 284.77 eV, 285.67 eV, and 288.87 eV (Zhou et al., 2026); Figure 1F exhibited O 1s splitting peaks corresponded to Fe-O, C-O/C-O=C, and H_2_O at the binding energy of 529.87 eV, 531.47 eV, and 533.27 eV (Zhou et al., 2026); Figure 1G revealed Ni 2p splitting peaks corresponded to Ni 2p_3/2_ and their satellite peaks at the binding energy of 854.97 eV, 856.37 eV, 860.87 eV, and 862.37 eV (Zeng X. Z. et al., 2026), and the distribution of the data points was somewhat scattered, which suggested the content of Ni in Ni_0.1_Mg_0.8_Cu_0.1_Fe_2_O_4_ MNPs was relatively small; Figure 1H displayed Mg 1s splitting peaks at the binding energy of 1302.97 eV, 1303.87 eV, and 1304.77 eV (Al-Quhali et al., 2026); Figure 1I demonstrated Cu 2p splitting peaks corresponded to Cu 2p_3/2_ and their satellite peaks at the binding energy of 933.77 eV, 934.77 eV, 941.27 eV, and943.77 eV (Halligudra et al., 2024), similar to the distribution of the data points for Ni in Ni_0.1_Mg_0.8_Cu_0.1_Fe_2_O_4_ MNPs, the content of Cu in the MNPs was also relatively small; while, Figure 1J showed the peaks observed at the binding energy of 711.07 eV and 724.67 eV, which corresponded to Fe 2p_3/2_ and Fe 2p_1/2_, respectively (Halligudra et al., 2021). Among them, the splitting peaks at 710.87 eV and 724.57 eV were attributed to Fe(II), while those at 713.07 eV and 727.67 eV belonged to Fe(III), respectively, along with two satellite peaks at 718.87 and 733.77 eV (Li et al., 2026). The nitrogen adsorption-desorption isotherms were shown in Figure 1K, and the pore size distribution was set into it. The adsorption and desorption curves did not overlap completely, indicating a typical type IV nitrogen adsorption–desorption isotherm, reflecting the mesoporous structure of Ni_0.1_Mg_0.8_Cu_0.1_Fe_2_O_4_ MNPs, which resulted in the large specific surface area. Their specific surface area reached 87.9 m^2^/g, and their pore size distributed from 3 nm–6 nm. These results revealed that Ni_0.1_Mg_0.8_Cu_0.1_Fe_2_O_4_ MNPs exhibited a larger adsorption capacity. The zeta potentials of Ni_0.1_Mg_0.8_Cu_0.1_Fe_2_O_4_ MNPs under different pH values are shown in Figure 1L. As the pH increased from 3 to 13, the zeta potential gradually decreased. When pH was in 3–9, the zeta potential was positive; when the pH exceeded 11, the zeta potential changed to negative.

### 3.2 Systematic exploration of synthetic variables for preparation of Ni_x_Mg_y_Cu_(1-x-y)_Fe_2_O_4_ MNPs formation

**FIGURE 2:**
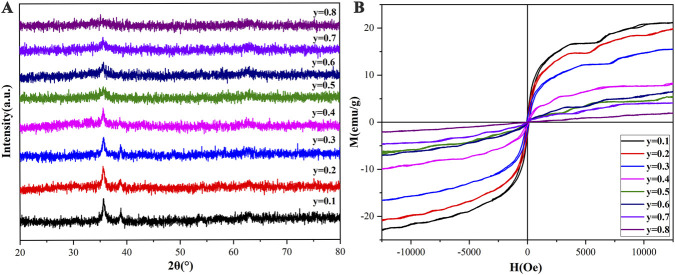
The XRD spectra **(A)** along with magnetic hysteresis curves **(B)** of Ni_0.1_Mg_y_Cu_(1-x-y)_Fe_2_O_4_ MNPs were derived from samples that were synthesized via calcination at 400 °C utilizing 25 mL of anhydrous ethanol.

To determine the optimal elemental ratio, XRD patterns of the synthesized Ni_x_Mg_y_Cu_(1-x-y)_Fe_2_O_4_ MNPs (with x = 0.1 and y ranging from 0.1 to 0.8) were examined (Figure 2A). As was well-known, the broader peaks in XRD patterns typically indicated MNPs with smaller dimensions and reduced crystallinity. As shown in Figure 2A, A non-linear trend in peak intensity was observed as x increased, with an initial decline followed by a subsequent rise, reflecting corresponding changes in the size and crystallinity of the MNPs. The average crystallite size (D) of the MNPs subsequently estimated using Scherrer’s formula, specified in Equation 6 (Chang et al., 2025).

$$D=\frac{k\lambda }{\beta \cos\theta }$$ (6)

The average crystallite size, represented as D (nm) in nanometers, was determined using the Scherrer equation, this calculation employed a shape factor (k) of 0.89, a wavelength (λ) of 0.154056 nm corresponding to Cu-Kα X-ray source, β was the FWHM for the diffraction peak expressed in radians, while θ indicated the Bragg angle as defined by Bragg’s law. Average grain sizes were evaluated for Ni_x_Mg_y_Cu_(1-x-y)_Fe_2_O_4_ MNPs (with x = 0.1 and y ranging from 0.1 to 0.8) were determined to be 24.6, 22.0, 19.7, 27.4, 21.3, 25.1, 27.5, and 17.4 nm, respectively. The hysteresis loops of these compositions, presented in Figure 2B, revealed that all samples exhibited typical soft magnetic behavior. As y increased from 0.1 to 0.8, corresponding to a rise in Mg content and a reduction in Cu content, the saturation magnetization decreased from 21.13 emu/g to 1.96 emu/g, mainly due to changes in elemental composition. In other words, variations in elemental composition could introduce different crystal defects, resulting in different magnetic properties and Ms values. Despite the reduction in magnetization, the composition with y = 0.8, which had the smallest crystallite size of 17.4 nm, was identified as optimal for applications requiring a high specific surface area.

**FIGURE 3:**
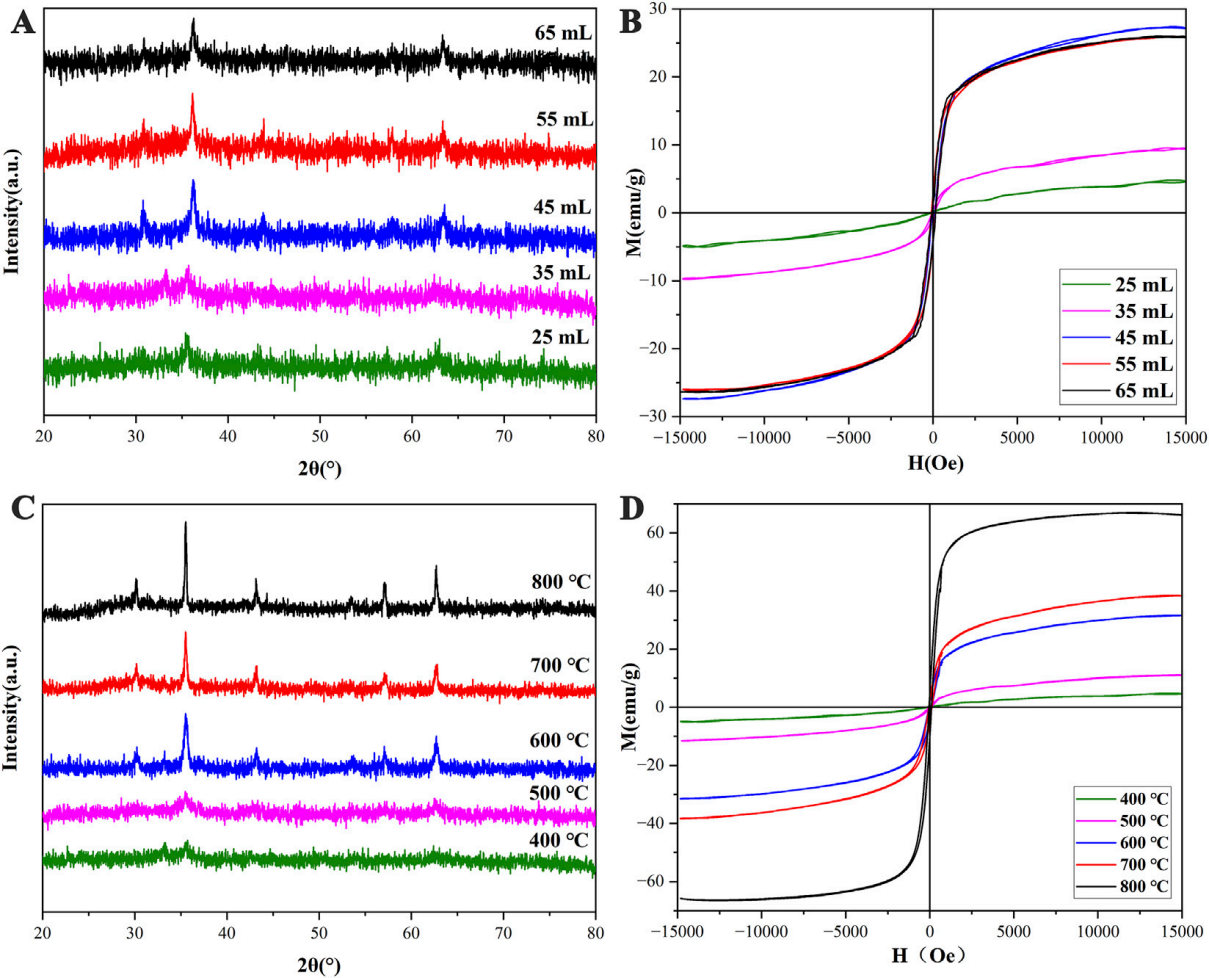
Characterization of Ni_0.1_Mg_0.8_Cu_0.1_Fe_2_O_4_ MNPs synthesized under different conditions. **(A,C)** XRD patterns and **(B,D)** M-H loops using varying quantities of anhydrous ethanol at multiple temperature settings while maintaining a constant reaction duration of 2 h.

The quantity of anhydrous ethanol used markedly influenced the crystallinity and magnetic properties of Ni_0.1_Mg_0.8_Cu_0.1_Fe_2_O_4_ MNPs synthesized at 400 °C. X-ray diffraction results, shown in Figure 3A, revealed distinct changes in major peak intensities as ethanol volumes ranged from 25 mL to 65 mL. These intensities increased up to 55 mL, then declined at 65 mL. Crystallite sizes, calculated with the Scherrer equation, followed a similar trend: 17.4 nm (25 mL), 19.2 nm (35 mL), 20.2 nm (45 mL), 24.3 nm (55 mL), and 21.5 nm (65 mL). Figure 3A displays the XRD patterns of these MNPs across varying ethanol amounts. The key peak at 35.53° intensified notably with rising solvent volume from 25 mL to 65 mL. This non-linear variation suggests a balance between enhanced material dispersion at higher ethanol levels and extended combustion time. As the ethanol volume increased from 25 to 55 mL, combustion promoted crystallite growth, whereas a further increase to 65 mL enhanced precursor dispersion, resulting in a reduction in crystallite size.

Figure 3B illustrates the hysteresis loops of Ni_0.1_Mg_0.8_Cu_0.1_Fe_2_O_4_ MNPs, synthesized with varying volumes of anhydrous ethanol. The saturation magnetization (M_s_) increased with increasing ethanol volume up to 45 mL, reaching a maximum of 27.30 emu/g, before decreasing at higher volumes, such as 65 mL. This trend suggests that the synthesis conditions significantly influenced the magnetic properties of the MNPs. While larger crystallite sizes were generally associated higher M_s_ due to reduced surface spin disorder, the observed peak in M_s_ at an intermediate ethanol volume indicated that additional factors, including crystallinity and surface defects, also played critical roles in shaping the magnetic behavior of these MNPs.

The influence of calcination temperature on the magnetic characteristics of Ni_0.1_Mg_0.8_Cu_0.1_Fe_2_O_4_ MNPs was carefully explored using X-ray diffraction (XRD) analysis (Figure 3C). As the calcination temperature increased from 400 °C to 800 °C in steps of 100 °C, the XRD peaks sharpened and intensified, indicating an enhancement in the crystallinity of the MNPs. The grain sizes, calculated using the Scherrer equation, exhibited a clear temperature-dependent trend: 17.4 nm at 400 °C, 20.0 nm at 500 °C, 26.7 nm at 600 °C, 29.1 nm at 700 °C, and 40.0 nm at 800 °C, also indicating the increase trend of grain growth with the increasing temperature. This phenomenon may be attributed to sintering or phase instability at the highest temperature, which could have inhibited further grain growth. These variations in grain size and crystallinity, driven by the calcination temperature, had significant implications for the adsorption properties of the Ni_0.1_Mg_0.8_Cu_0.1_Fe_2_O_4_ MNPs. The enhanced crystallinity and optimized grain size at intermediate calcination temperatures were expected to improve the material’s electrochemical performance, particularly in terms of capacitance, conductivity, and adsorption capacity.

The structural observations were further supported by magnetic characterization as presented in Figure 3D. This analysis revealed that the M_s_ generally increased with calcination temperature, consistent with the expected trend wherein larger crystallite sizes resulted in higher M_s_ due to reduced surface spin disorder. The optimal synthesis conditions, considering both structural and magnetic properties, were identified as 25 mL of anhydrous ethanol and calcination at 400 °C for 2 h. In this experimental configuration, the MNPs exhibited a crystallite size of 17.4 nm and an M_s_ of 4.73 emu/g, achieving an effective compromise between high surface area and adequate magnetic responsiveness. The selection of a 25 mL ethanol volume proved particularly advantageous for adsorption applications, as it enhanced surface area while preserving sufficient magnetic separability.

### 3.3 Adsorption kinetics

The kinetic behavior of MB uptake by the Ni_0.1_Mg_0.8_Cu_0.1_Fe_2_O_4_ MNPs was investigated by fitting empirical data to three widely utilized kinetic models. These models are commonly employed in adsorption studies to characterize the dynamic process and identify the predominant rate-controlling steps. As presented in Equation 7 (Raganati et al., 2025), the pseudo-first-order model posits that the adsorption rate is directly proportional to the difference between the equilibrium adsorption capacity (q_e_) and the adsorption capacity at a given time (q_t_). Conversely, the pseudo-second-order model, represented by Equation 8 (Abdul Nishad et al., 2024), is typically associated with chemisorption mechanisms and assumes that the adsorption rate depends on the square of the difference between q_e_ and q_t_. Additionally, the Weber–Morris intraparticle diffusion model, described by Equation 9 (Zhao M. et al., 2024), assesses the influence of MB diffusion within the internal pores of the MNPs on the overall adsorption kinetics.

$$\ln\left(q_{t}-q_{e}\right)=\ln q_{e}-K_{1}t$$ (7)

$$q_{t}=\frac{K_{2}{q_{e}}^{2}t}{1+K_{2}q_{e}t}$$ (8)

$$q_{t}=C_{i}+k_{i}t^{0.5}$$ (9)

where, q_e_ (mg/g) represented the quantity of MB captured by Ni_0.1_Mg_0.8_Cu_0.1_Fe_2_O_4_ MNPs at equilibrium conditions; q_t_ (mg/g) denoted the amount of MB adsorbed onto the MNPs, q_t_ was the adsorption capacity at time t (min^−1^); K_1_ (g/mg·min), K_2_ (g/mg·min), and k_i_ (mg/g·h^1/2^) represented the sorption rate constants for the respective adsorption kinetics models; C_1_ was determined based on the boundary layer thickness.

**FIGURE 4:**
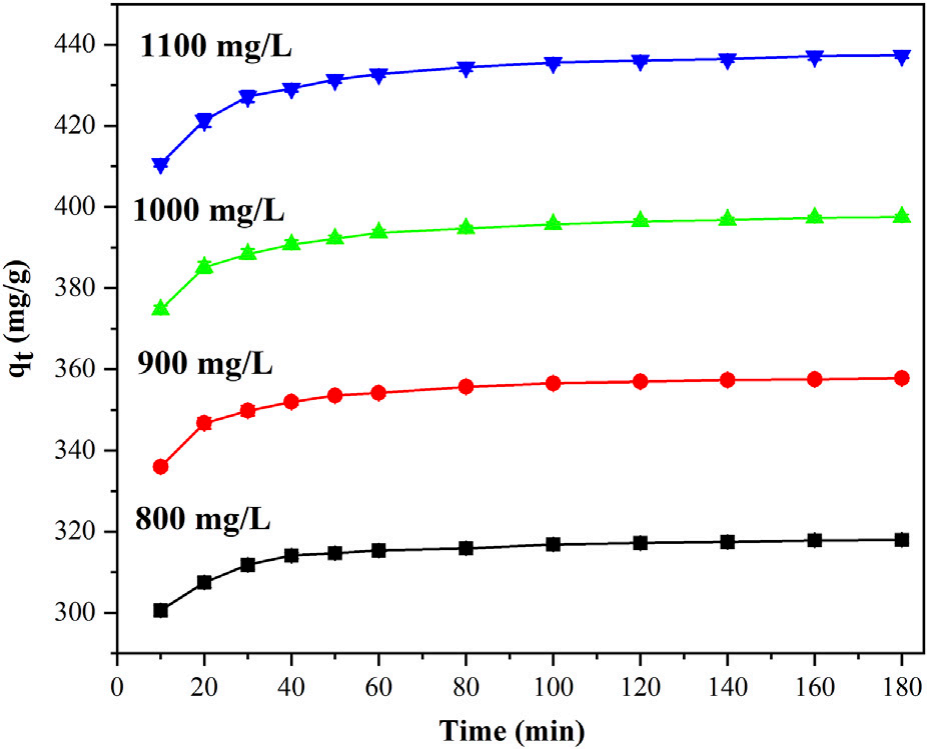
Sorption results of MB at different starting concentrations on Ni_0.1_Mg_0.8_Cu_0.1_Fe_2_O_4_ MNPs prepared at 400 °C for 2 h with 25 mL of anhydrous ethanol.

MB adsorption kinetics onto Ni_0.1_Mg_0.8_Cu_0.1_Fe_2_O_4_ MNPs were studied at initial concentrations of 800, 900, 1,000, and 1100 mg/L, reaching equilibrium within approximately 80 min for all tested concentrations shown in Figure 4. The enormous equilibrium adsorption capacities (q_e_) amounted to 317.58 mg/g for 800 mg/L, 367.35 mg/g for 900 mg/L, 396.65 mg/g for 1,000 mg/L, and 436.45 mg/g for 1,100 mg/L.

**FIGURE 5:**
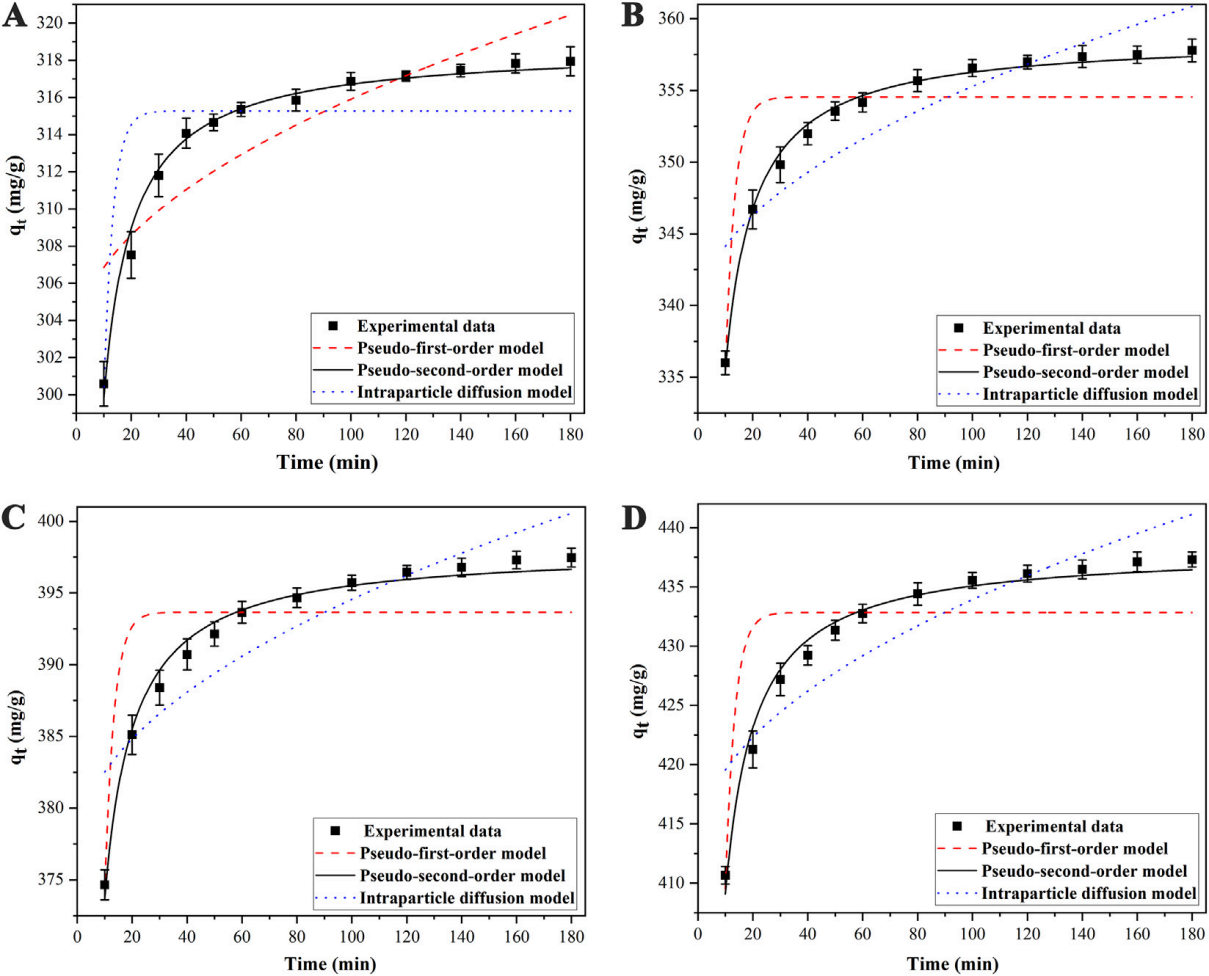
Adsorption kinetics of MB on Ni_0.1_Mg_0.8_Cu_0.1_Fe_2_O_4_ MNPs calcined at 400^o^C for 2 h with initial MB concentrations of 800 mg/L **(A)**, 900 mg/L **(B)**, 1,000 mg/L **(C)**, and 1,100 mg/L **(D)**.

The adsorption kinetics of methyl blue (MB) onto Ni_0.1_Mg_0.8_Cu_0.1_Fe_2_O_4_ MNPs were analyzed using the pseudo-first-order, pseudo-second-order, and intraparticle diffusion models. The outcomes of these model fittings for different initial MB concentrations are displayed in Figure 5. Specifically, Figure 5 illustrated the intraparticle diffusion plots, which exhibited multi-linear behavior, indicating that multiple processes influenced the adsorption kinetics. The kinetic parameters and adjusted R-squared (Adj R^2^) values for each model were provided in Table 1. From the data, it was evident that the pseudo-second-order model provided the best fitting to the experimental data, with Adj R^2^ values ranging from 0.9824 to 0.9944 across tested MB concentrations. In contrast, the pseudo-first-order model yielded lower Adj R^2^ values between 0.6419 and 0.7130. However, Adj R^2^ values for the intraparticle diffusion model ranged from 0.6697 to 0.7558. The substantially elevated Adj R^2^ in the pseudo-second-order model pointed to its superior accuracy in depicting MB adsorption on Ni_0.1_Mg_0.8_Cu_0.1_Fe_2_O_4_. Fitting the pseudo-second-order kinetics model revealed a main chemisorption process, featuring valence forces from electron sharing or exchange between adsorbent and adsorbate. This finding was consistent with the proposed interactions between MB molecules and the surface functional groups of Ni_0.1_Mg_0.8_Cu_0.1_Fe_2_O_4_ MNPs.

**TABLE 1:** MB adsorption kinetics parameters on Ni_0.1_Mg_0.8_Cu_0.1_Fe_2_O_4_ at varying initial concentrations and ambient temperature at varying initial concentrations and ambient temperature.

Kinetics model	Parameter	MB concentration values at the start (mg/L)			
		800	900	1,000	1,100
Pseudo-first-order	R^2^	0.7326	0.7391	0.7001	0.6745
	K_1_	0.3020	0.2909	0.2989	0.2920
	Adj. R^2^	0.7059	0.7130	0.6701	0.6419
Pseudo-second-order	R^2^	0.9885	0.9949	0.9873	0.9840
	K_2_	0.0050	0.0040	0.0040	0.0032
	Adj. R^2^	0.9873	0.9944	0.9861	0.9824
Weber–Morris internal diffusion	R^2^	0.6998	0.7404	0.7770	0.7780
	K_i_	1.3245	1.6311	1.7602	2.1031
	Adj. R^2^	0.6697	0.7145	0.7547	0.7558

### 3.4 Adsorption isotherm

Three standard isotherm models—Langmuir, Freundlich, and Temkin—were used to study MB adsorption equilibrium on Ni_0.1_Mg_0.8_Cu_0.1_Fe_2_O_4_ MNPs. The Langmuir approach, premised on monolayer formation, helped in quantifying the adsorption. The Langmuir adsorption isotherm model, which assumed homogeneous surface with monolayer adsorption, was utilized to quantify the adsorption process. In this model, the adsorbent surface is thought to possess a specific number of identical sites, each able to attach to a single molecule, with no interactions among the adsorbed molecules and uniform across all sites. Consequently, the Langmuir model the Langmuir model characterizes monolayer formation by the adsorbate across the adsorbent surface with consistent adsorption heat. The corresponding isotherm equation was presented in Equation 10 (Diksha et al., 2025).

The Freundlich adsorption isotherm model served as a valuable empirical tool for describing adsorption on heterogeneous surfaces, particularly in cases involving multilayer adsorption. In contrast to the Langmuir model, it did not impose a saturation limit and accounted for variations inside energies, rendering it applicable to a diverse array of environmental and industrial contexts. The empirical equation, presented in Equation 11 (Gentile et al., 2025), enabled the fitting of experimental data, though it exhibited limitations at elevated pressures. The model characterized an exponential increase in adsorption capacity. By comparison, the Temkin adsorption isotherm model incorporated accounted for how temperature influenced the adsorption process.

As per the Temkin isotherm framework, key interplay occurs among the adsorbing material and the substance being adsorbed. Based on the Temkin adsorption isotherm model, the adsorption enthalpy decreases linearly as surface coverage increases, resulting from interactions between the adsorbate and the adsorbent. This model accounted for adsorbate–adsorbent interactions and was applicable to intermediate concentration ranges, avoiding extremes where the assumptions might not hold. The model proposed a straight-line reduction in adsorption enthalpy as surface occupancy increases, unlike alternative isotherms that typically presume a log-based correlation. The corresponding equation was presented in Equation 12 (Ling et al., 2022; Zhang et al., 2026).

$$q_{e}=\frac{q_{\max}K_{L}C_{e}}{1+K_{L}C_{e}}$$ (10)

$$q_{e}=K_{F}C_{e}^{\frac{1}{n}}$$ (11)

$$q_{e}=B_{T}\ln\left(A_{T}C_{e}\right)$$ (12)

$$B_{T}=R_{g}T/K_{T}$$ (13)

where, q_e_ (mg/g) stood for the balanced uptake amount of MB by Ni_0.1_Mg_0.8_Cu_0.1_Fe_2_O_4_ MNPs; q_max_ (mg/g) denoted the peak sorption capacity; K_L_ represented the equilibrium constant for Langmuir-based modeling, in contrast to K_F_ signified the Freundlich adsorption isotherm; while the parameter n indicated the strength of adsorption; while C_e_ (mg/L) indicates the final adsorbate level in the aqueous environment post-equilibrium. Regarding Temkin specifics A_T_ (L/g) respectively stood for the equilibrium, B (J/mol) related to sorption thermal variations, R represented the standard gas constant at 8.314 J/(mol·K), T (K) signifies temperature in absolute units, and K_T_ (J/mol) linked to the energetic aspects of the sorption mechanism.

**FIGURE 6:**
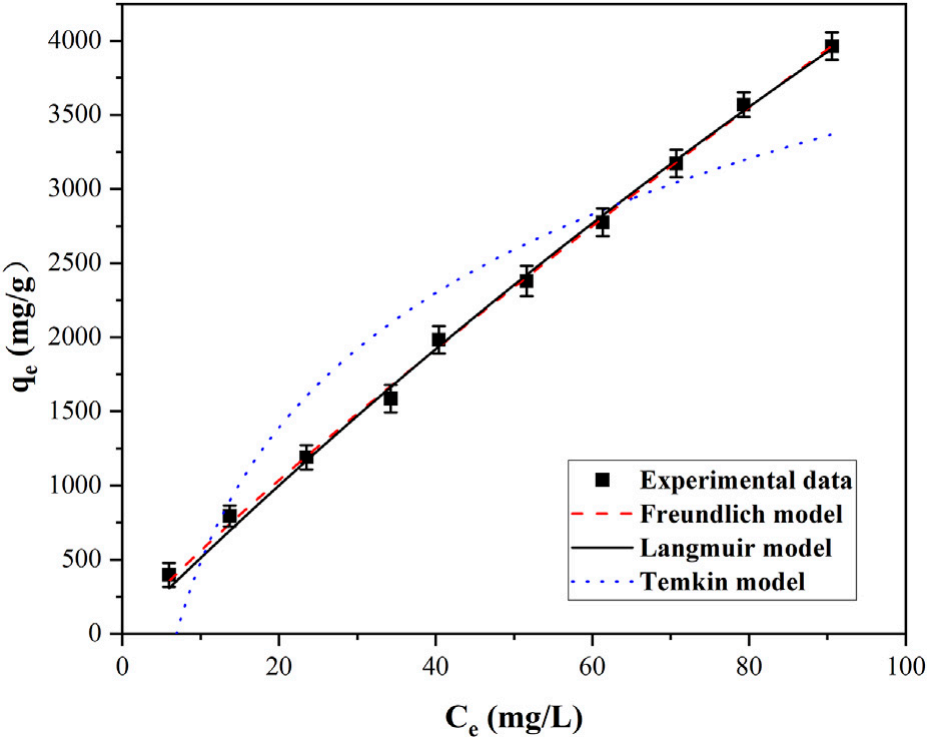
Illustration of the isotherm profile depicting MB uptake by Ni_0.1_Mg_0.8_Cu_0.1_Fe_2_O_4_ MNPs at 400 °C for 2 h with 400–4,000 mg/L.

The adsorption data were analyzed using three isotherm models, with their fitted curves displayed in Figure 6 and parameters listed in Table 2. MB adsorption onto Ni_0.1_Mg_0.8_Cu_0.1_Fe_2_O_4_ MNPs was most effectively described by the Freundlich equation (Adj. R^2^ = 0.9893) compared to the Langmuir (Adj. R^2^ = 0.9838) and Temkin (Adj. R^2^ = 0.9759) models. The data suggested that the adsorption mechanism was likely multilayered, which suggested that the chemical adsorption was accompanied by a small amount of physical adsorption. The 1/n value, indicative of the adsorption intensity, was calculated as 0.7967, suggesting a favorable adsorption process. These results confirmed that the Ni_0.1_Mg_0.8_Cu_0.1_Fe_2_O_4_ MNPs were effective for dye removal. The observed increasing MB uptake at elevated initial concentrations, as shown in Figure 6, might be linked to the improved attachment of MB molecules to the MNPs. This was concluded that the Ni_0.1_Mg_0.8_Cu_0.1_Fe_2_O_4_ MNPs were well-suited for eliminating dyes from wastewater systems.

**TABLE 2:** Isotherm parameters from langmuir, freundlich, and temkin models for the adsorption of MB onto Ni_0.1_Mg_0.8_Cu_0.1_Fe_2_O_4_ at 25 °C.

Iostherm model	Adj.R^2^	Parameter	Value
Langmuir	0.9838	q_max_	4510.0057
		K_L_	0.0153
Freundlich	0.9893	K_F_	94.6826
		1/n	0.7967
Temkin	0.9759	B_T_	681.7908
		A_T_	0.2523

### 3.5 Consequences of pH changes for sorption and recycle of Ni_0.1_Mg_0.8_Cu_0.1_Fe_2_O_4_ MNPs

**FIGURE 7:**
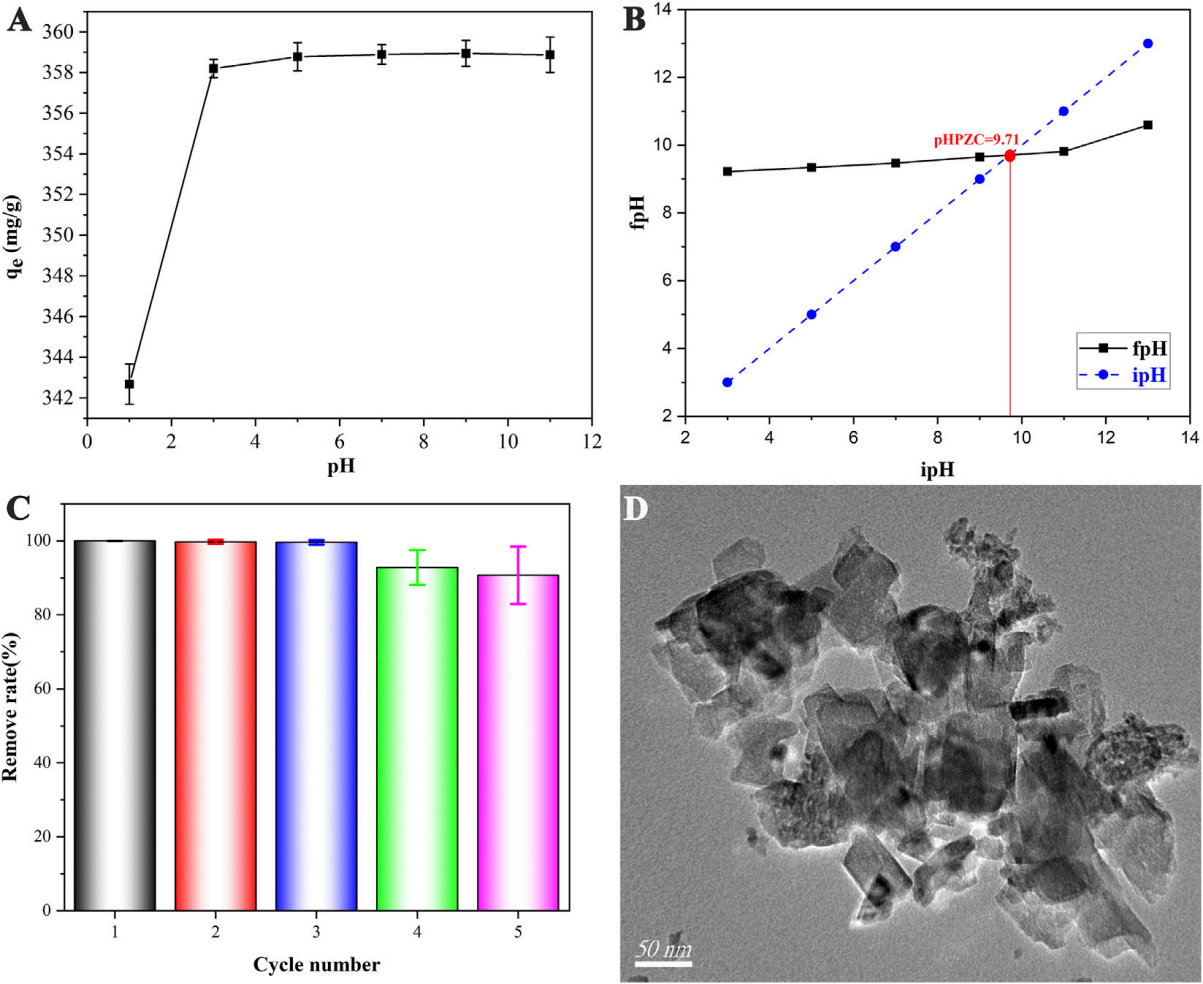
Effects of pH levels on Ni_0.1_Mg_0.8_Cu_0.1_Fe_2_O_4_ MNPs uptake capability for MB **(A)**, pH at the point of zero charge (pHPZC) **(B)**, recycle performance of Ni_0.1_Mg_0.8_Cu_0.1_Fe_2_O_4_ MNPs **(C)**, and TEM of five-recycled Ni_0.1_Mg_0.8_Cu_0.1_Fe_2_O_4_ MNPs **(D)**.

**FIGURE 8:**
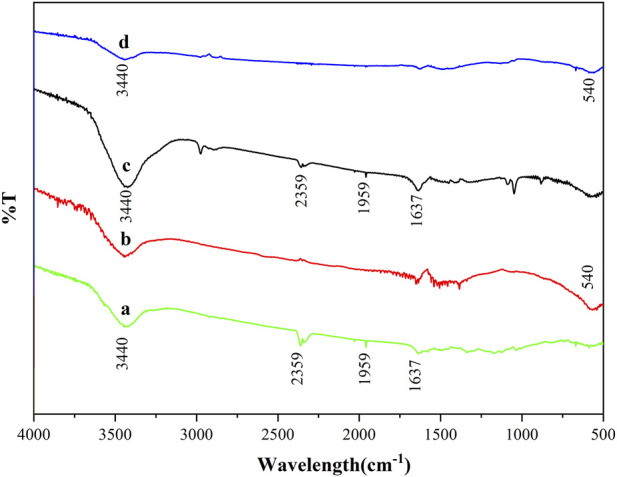
MB spectrum FT-IR **(a)**, Ni_0.1_Mg_0.8_Cu_0.1_Fe_2_O_4_ MNPs **(b)**, Ni_0.1_Mg_0.8_Cu_0.1_Fe_2_O_4_ MNPs absorbed MB **(c)**, the recalcined Ni_0.1_Mg_0.8_Cu_0.1_Fe_2_O_4_ MNPs **(d)**.

The capacity for regeneration and recovery of adsorbents was a critical criterion when gauging their uptake efficiency. There were many influence factors on the adsorption, including solution pH, ionic strength, temperature, competing ions, and so on. Among them, the effect of solution pH was one of largest factors. The uptake efficiency of MB by Ni_0.1_Mg_0.8_Cu_0.1_Fe_2_O_4_ MNPs showed a marked dependence on the acidity level of the dye mixture. As shown in Figure 7A, adsorption capacity showed a gradual increase as pH was raised from 1 to 3. This trend might result from possible modifications in MB molecules at different pH levels. Beyond a pH of 3, the adsorption capacity of Ni_0.1_Mg_0.8_Cu_0.1_Fe_2_O_4_ MNPs for MB stabilized and showed minimal fluctuations. Specifically, at an initial 400 mg/L MB concentration, the maximum adsorption capacity was recorded at 358.94 mg/g when the pH surpassed 9, indicating that all adsorption sites on the Ni_0.1_Mg_0.8_Cu_0.1_Fe_2_O_4_ MNPs were fully saturated. These results suggested that modifying the pH of the solution is an effective means of regulating the adsorption capacity of Ni_0.1_Mg_0.8_Cu_0.1_Fe_2_O_4_ MNPs for MB,while also promoting the release of MB through pH adjustments. Figure 7B revealed the final pH (fpH) of Ni_0.1_Mg_0.8_Cu_0.1_Fe_2_O_4_ MNPs, obviously, fpH increased with the rise of pH value, and fpH values were against the initial pH (ipH) values; while, the pH at the point of zero charge (pHPZC) reached 9.71 where fpH was equal to ipH. All the results indicated that Ni_0.1_Mg_0.8_Cu_0.1_Fe_2_O_4_ MNPs maintained a stable positive charge across a wide pH range. The recycling performance of Ni_0.1_Mg_0.8_Cu_0.1_Fe_2_O_4_ MNPs for MB removal was demonstrated in Figure 7C. The removal rate decreased as the number of cycles increased, a trend likely attributable to enhanced sintering of the MNPs, a resulting in pore collapse and a reduction in specific surface area. Despite this decline, the Ni_0.1_Mg_0.8_Cu_0.1_Fe_2_O_4_ MNPs retained 90.7% of their initial removal rate after 5 cycles, underscoring their excellent recyclability. This superior recyclability highlighted their significant potential for dye removal applications. A comparative analysis of MB adsorption onto various adsorbents was summarized in Table 3. Compared with other magnetic adsorbents and the conventional adsorbents, the adsorption capacity of MB onto Ni_0.1_Mg_0.8_Cu_0.1_Fe_2_O_4_ MNPs that was 2–44.7 times higher than that of the other adsorbents. Furthermore, their superior recycling capacity and broader pH resistance range (3–11) distinguished them among adsorbents for MB adsorption. The TEM image of the Ni_0.1_Mg_0.8_Cu_0.1_Fe_2_O_4_ MNPs after five adsorption–desorption cycles is shown in Figure 7D. The average particle size increased after repeated regeneration, which likely contributed to the decrease in adsorption capacity. FT-IR spectroscopy was employed to investigate the adsorption of MB onto Ni_0.1_Mg_0.8_Cu_0.1_Fe_2_O_4_ MNPs, as illustrated in Figure 8. The recorded spectrum revealed peaks at 1,637 cm^−1^, 1,959 cm^−1^, and 2,359 cm^−1^, representing typical markers for the absorption traits of MB. Additionally, curve b exhibited a prominent peak at 1,579 cm^−1^, denoting a sturdy Fe–O linkage. In curve c, both the Fe–O bond and MB characteristic peaks were observed, indicating successful adsorption of MB onto the surfaces of Ni_0.1_Mg_0.8_Cu_0.1_Fe_2_O_4_ MNPs. Finally, in curve d, MB had been removed from the surface of the Ni_0.1_Mg_0.8_Cu_0.1_Fe_2_O_4_ MNPs. These results indicated the potential for regeneration of Ni_0.1_Mg_0.8_Cu_0.1_Fe_2_O_4_ MNPs via calcination at high temperatures.

**TABLE 3:** Comparison of different adsorbents for MB removal and Ni_0.1_Mg_0.8_Cu_0.1_Fe_2_O_4_ MNPs.

Adsorbent	q_max_ (mg/g)	References
SF-N-CDs	620	Yu et al. (2025)
Fe_3_O_4_@SiO_2_@MBH-CR	959	Cui et al. (2025)
AELS	153	Huo et al. (2024)
Cu_0.1_Mn_0.9_Fe_2_O_4_ MNPs	100.94	Sun et al. (2026)
Co_0.8_Cu_0.2_Fe_2_O_4_ MNPs	138.2	Yin et al. (2021)
Ni_0.4_Mg_0.3_Zn_0.3_Fe_2_O_4_ MNPs	707.1	Yu et al. (2020)
Ni_0.5_Cu_0.5_Fe_2_O_4_ MNPs	131.64	Pan et al. (2019)
Co_0.1_Mg_0.7_Cu_0.2_Fe_2_O_4_ MNPs	2255.7	Zhang et al. (2026)
Ni_0.1_Mg_0.8_Cu_0.1_Fe_2_O_4_ MNPs	4510	This work

### 3.6 Thermodynamics

Thermodynamic analysis was performed to investigate the effect of temperature on the adsorption of MB onto Ni_0.1_Mg_0.8_Cu_0.1_Fe_2_O_4_ MNPs. Variations in Gibbs free energy (ΔG) served to quantify the inherent spontaneity within the sorption mechanism. ΔG values for MB adsorption on Ni_0.1_Mg_0.8_Cu_0.1_Fe_2_O_4_ MNPs were calculated as −12.38 kJ/mol under 303 K, −11.83 kJ/mol at 313 K, and −10.81 kJ/mol for 323 K (Table 4). The negative ΔG values indicated that the adsorption of MB onto Ni_0.1_Mg_0.8_Cu_0.1_Fe_2_O_4_ MNPs occurred spontaneously. Meanwhile, the adsorption enthalpy change (ΔH) of −36.01 kJ/mol indicated an exothermic process, and the tendency for spontaneous heat release was greater at 303 K. The entropy of adsorption (ΔS) of −77.74 J/(molK) suggested the disorder of the adsorption system decreases. The reason for this was that, during the adsorption process, MB molecules were chemically adsorbed onto the MNPs, replacing the disordered water molecules on the MNPs surface and thereby forming a more ordered adsorption system composed of MB and Ni_0.1_Mg_0.8_Cu_0.1_Fe_2_O_4_ MNPs. Furthermore, the relatively large negative ΔS value indicates that the ordering effect associated with MB adsorption outweighed the increase in molecular volume caused by the replacement of water molecules. Therefore, thermodynamic analysis indicated that the adsorption of MB onto the MNPs was a spontaneous and exothermic process with an increase in orderliness.

**TABLE 4:** Thermodynamic factors involved in MB sorption onto Ni_0.1_Mg_0.8_Cu_0.1_Fe_2_O_4_ MNPs.

*T*(K)	*K*_d_	Δ*G* (kJ/mol)	Δ*H* (kJ/mol)	Δ*S* (J/(mol·K))
303	136.27	−12.38	−36.01	−77.74
313	94.44	−11.84		
323	56.13	−10.82		

## 4 Conclusion

Ni_0.1_Mg_0.8_Cu_0.1_Fe_2_O_4_ MNPs were synthesized via rapid combustion method and evaluated for the adsorption and removal of MB. The synthesis conditions were optimized to achieve desirable physicochemical properties. MNPs were prepared by optimizing the elemental composition, calcination temperature, and volume of absolute ethanol, with 25 mL of alcohol and calcination at 400 °C for 2 h. The optimized MNPs exhibited a spherical morphology with an average particle size of approximately 17.4 nm and a saturation magnetization of 4.73 emu/g. These characteristics enhanced their adsorption capacity and separation efficiency.

Adsorption experiments showed that MB uptake followed pseudo-second-order rate laws and Freundlich modeling, suggesting a chemisorption process involving multiple layers. Evaluations of thermodynamic properties confirmed spontaneous adsorption without extra energy input. The MNPs maintained high adsorption performance over a wide pH range (3–11) and exhibited excellent stability under different pH and ionic conditions, making them promising adsorbents for dye wastewater treatment. After five cycles, the MNPs retained 90.7% of their initial adsorption capacity, indicating excellent reusability. All the results demonstrated high adsorption efficiency, environmental adaptability, reusability, and promising applications of the synthesized MNPs for the dye wastewater treatment. Future studies should investigate factors affecting magnetic separation efficiency, production cost, and environmental safety, particularly the potential leaching of metal ions from ferrite nanoparticles during the treatment of real wastewater. Such studies will provide more practical and fundamental data to support their large-scale application in wastewater treatment.

## Data Availability

Publicly available datasets were analyzed in this study. This data can be found here: none.
